# Gender-based violence during COVID-19 lockdown: case study of a community in Lagos, Nigeria

**DOI:** 10.4314/ahs.v22i2.10

**Published:** 2022-06

**Authors:** Ojima Zechariah Wada, David Bamidele Olawade, Aminat Opeyemi Amusa, Jedidah Oluwadamisi Moses, Glory Jessica Eteng

**Affiliations:** 1 Division of Sustainable Development, College of Science and Engineering, Hamad Bin Khalifa University, Doha, Qatar; 2 Faculty of Public Health, University of Ibadan, Ibadan, Nigeria; 3 Department of Medicine and Surgery, University of Ibadan, Ibadan, Nigeria; 4 Advocating the Girl Child Organization, Nigeria

**Keywords:** Gender-based violence, COVID-19, women, girls, assault

## Abstract

**Background:**

Gender-based violence (GBV) has been identified to be one of the ripple effects of the global pandemic. In countries like Nigeria, the situation is hypothesized to be worse because of widespread poverty and gender inequalities.

**Objective:**

To examine the exposure of females to GBV during the first 3 months of the COVID-19 lockdown.

**Method:**

This cross-sectional study was conducted in a low-income community in Lagos. Semi-structured questionnaires were administered to 130 respondents selected via systematic random sampling.

**Results:**

The mean age of the respondents was 26.89 ± 8.67 years. Majority worked informal jobs, while only 50% had attained beyond primary education. Within the period, the respondents had been subjected to sexual (54.6%), physical (52.3%), verbal assault (41.5%), and online sexual harassment (45.4%); of which only 30% reported to the police. Furthermore, respondents subjected to sexual (p=0.004) and physical assault (p=0.032) during the period earned significantly less money than other respondents.

**Conclusion:**

The fact that over 1 out of every 2 females was subjected to at least one form of GBV within the short timeframe shows how unsafe girls and women in low-income communities are. This calls for proactive community-level interventions to curb the GBV menace.

## Introduction

Gender-based violence (GBV) is a public health menace that is highly prevalent across the globe. It consists of sexual abuse, child marriage, female genital mutilation, physical abuse, psychological abuse, and social media-based violence 1,2. According to the World Health Organization, around 1 in every 3 women globally has been subjected to either sexual or/and physical assault in their lifetime and some of the factors that predispose women to GBV are lack of education, male privilege, cultural and attitudinal constrains and societal relegation of women 3. Similar statistics have been reported in Nigeria, where around 1 in every 3 females between ages 15 and 49 were revealed to have been subjected to sexual assault 4. Other local surveys have reported a higher prevalence of GBV ranging from 42.3% to 89%, with psychological violence reported to be the most prevalent form of GBV^5^–^8^. Although there are cases of men experiencing some forms of GBV, women are disproportionately affected 9.

Women and girls have been identified to be even more vulnerable to GBV during moments of social disruption and insecurity. A survey among thousands of internally displaced persons in northern Nigeria reported that around a third and a fifth had been subjected to sexual and physical violence, respectively since their displacement 10. The COVID-19 pandemic has been reported to have ripple effects on GBV, particularly in areas where insecurity and social inequality are persistent 11. Due to the social construct in countries like Nigeria, women and girls are more susceptible to the economic impact of the pandemic, thereby making them more susceptible to GBV during the pandemic. A survey in Borno State reported that the pandemic had adversely affected the livelihoods of more women (71%) compared to men (51%). This was because the informal sector majorly comprises female workers and during the pandemic, most of the informal work sphere were closed due to lockdown restrictions 12. Within two weeks after the COVID-19 lockdown was enacted in Nigeria, there was a 56% increase in cases of GBV 13. In Lagos State, there was a three-fold increase in the cases of domestic and sexual violence reported via the hotlines within a month of the lockdown. The significant increase in the rate of GBV during the pandemic was also reported in countries like Canada, UK, USA, China, Germany, and Argentina, which is why GBV during COVID-19 era was referred to as the Shadow Pandemic 11,13,14

Due to the unprecedented rise in the cases of GBV during the pandemic, the Government declared a State of Emergency on rape and GBV in Nigeria 15. However, to effectively tackle this menace, it is important to assess the most prevalent forms of GBV and the available community structures designed to curb the menace at the community level. This research was conducted to determine the prevalence of GBV and the available support structures in a low-middle income community in Lagos State.

## Methods

The study was conducted in Eyin-Ogun Mafoluku, a low-income community in Oshodi Lagos State, Southwestern Nigeria. The study population consisted of females between the ages of 14 to 40 years old residing in the community. The survey was cross-sectional, solely utilizing a semi-structured questionnaire. The sample size (N) was obtained via the formula:

Where p was 50% due to the absence of data on the local prevalence of GBV, q was 50% (100-p), allowable error l was 10%

N= 4 × 50 × 50/102 = 100 young female respondents

Due to the peculiarity of the COVID-19 era, a 30% non-response rate was added to ensure all the respondents were reached. A total of 130 young female respondents participated in this survey.

Data was obtained in June 2020 when the lockdown lifted via house-to-house survey within the study community. The research enumerators were kitted with their Personal Protective Equipment (PPE) and observed all COVID-19 regulations during data collection process. The questionnaire was designed to obtain the respondents' socio-demographic data, exposure to GBV during the COVID-19 lockdown and the available community structures to protect against GBV.

### Data management and analysis

In this study, sexual assault constituted of at least an incidence of rape, attempted rape, molestation, and unconsented fondling while online sexual harassment referred to unwelcome sexual advances via social media platforms. Physical assault constituted of bodily harm or injury with or without a weapon, while verbal assault comprised of at least an incidence of spoken remarks made to threaten, elicit harm, or provoke fear. The data obtained was analyzed using SPSS version 20. The results were presented using descriptive statistics like measures of frequency and measures of proportion, and inferential statistics like independent t-test and logistic regression at 5% level of statistical significance.

### Ethical consideration

Ethical approval was obtained from Ekiti State University Teaching Hospital Ethics and Research Committee. All the respondents voluntarily participated in this survey. There was no form of coercion or undue compensation. Consent was obtained from guardians of respondents under 18 years of age. The privacy of every respondent was protected, no identifiable information was obtained, and the questionnaires were kept in a secured safe after data entry.

## Results

The respondents age ranged from 14 years to 40 years, with a mean age of 26.89 ± 8.67 years. The respondents' mean monthly income was 3,400 ± 1,577.95 Naira (ap-proximately USD 9), with a maximum income of 6,000 Naira (approximately USD 16). Less than a quarter of the respondents had acquired tertiary education while majority (64%) were single/cohabiting. Around a fifth of the respondents were unemployed, while the remainder were into informal jobs like hairdressing, trading, cooking, and fashion designing. Table 1 provides details about the respondents' sociodemographic characteristics.

**Table 1:** Socio-demographic characteristics of respondents

**Age**		
Less than 18	28	21.5
18 to 29	42	32.3
30 to 40	60	46.2
**Where do you stay**		
With parents	30	23.1
With relatives	36	27.7
With friends	37	28.5
Personal place	27	20.8
**Level of Education**		
Primary	27	20.8
Secondary	37	28.5
Tertiary	28	21.5
No formal education	38	29.2
**Marital status**		
Single	46	35.4
Married	47	36.2
Co-habiting	37	28.5
**Occupation**		
Unemployed	28	21.5
Hairdresser	30	23.1
Trader	23	17.7
Fashion designer	20	15.4
Cook/baker	29	22.3
**Family structure**		
Monogamous	67	51.5
Polygamous	63	48.5
Ethnicity		
Yoruba	36	27.7
Igbo	52	40.0
Hausa	42	32.3
**Religion**		
Christian	40	30.8
Islam	38	29.2
Traditionalist	27	20.8
Atheist	25	19.2

### Prevalence of GBV during the COVID-19 Pandemic

**Figure 1:**
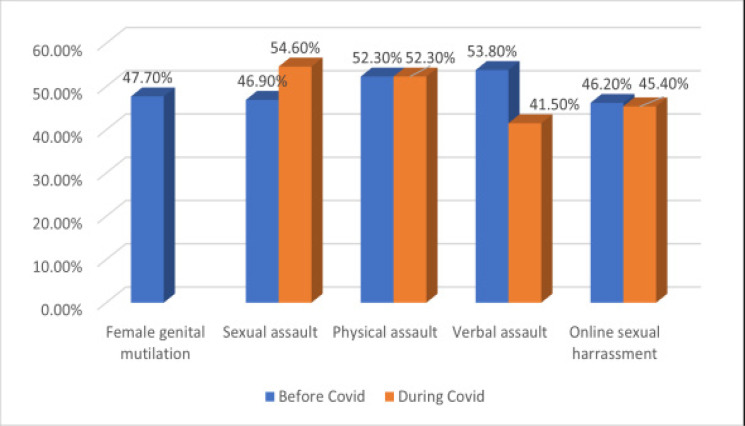
Prevalence of various forms of GBV before and during COVID-19

Data relating to the exposure of respondents to the various forms of GBV before the pandemic and during the pandemic is represented in Figure 1. The respondents reported a 7.7% increase in cases of sexual assault during the pandemic compared to pre-COVID-19 period. Over half of the respondents were subjected to physical and sexual assault during the pandemic, while over 40% experienced verbal assault and online sexual harassment during the pandemic. Also, just less than half of the respondents had been subjected to female genital mutilation (FGM).

**Figure 2:**
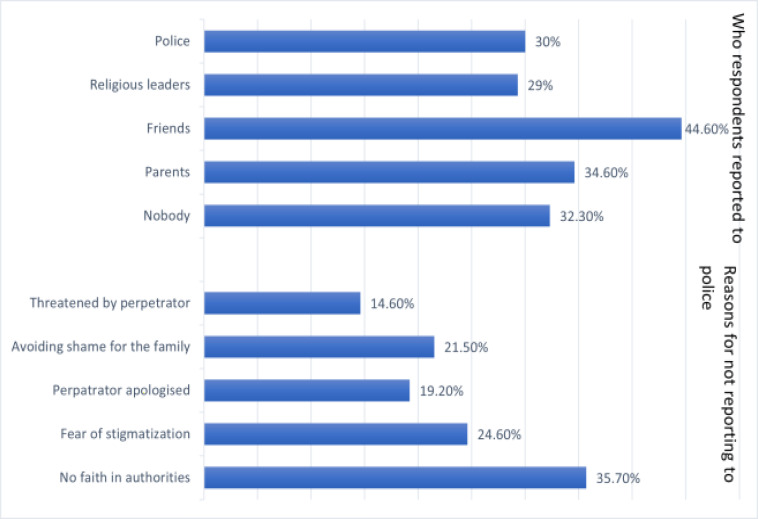
Common people GBV victims reported to and reasons for not reporting to the police

Furthermore, less than one-third of the respondents revealed that they reported to the police after being assaulted. The most prominent reason why the respondents did not report GBV cases to the appropriate security agencies was because they did not have faith in the authorities. Figure 2 provides details about the common people respondents reported incidences of GBV to, as well as the common reasons why they did not report to the police.

### Community structures to protect against GBV

Examining the community, there were no community-specific structures to protect women and girls against GBV. The only structures available was the State hotline to report cases of physical and sexual abuse and the local police. However, there were no informative posters revealing the hotline to reach out to when GBV cases arise. Just over half (52.3%) of the respondents were aware of available structures put in place to protect girls and women from physical and sexual abuse. Moreover, most of the respondents (56.2%) reported that they had no confidence in the authorities to advocate for the rights of girls and women in the society. Most of the respondents also revealed that they did not feel females were well protected from all forms of abuse in their society (59.2%) and that females were mostly blamed in cases associated with rape (53.1%). Furthermore, only half of the respondents felt there was enough awareness about rape and sexual assault in the society.

### Association between forms of GBV and the respondents' income level/age

There were statistically significant associations derived between the income level of the respondents and their subjection to sexual (p=0.004) and physical assault (p=0.032). Respondents that had been subjected to sexual assault and physical assault earned significantly lower on the average every month compared to those that earned higher. However, there were no statistically significant association between respondents' income level and the occurrence of verbal assault (p=0.083) and online harassment (p=0.099). Furthermore, there were no statistically significant associations derived between the respondents' age and the occurrence of the selected forms of GBV. Table 2 provides details of the various associations.

**Table 2:** Association between respondents' age/average income and GBV

	Sexual assault				
	t	f	df	Mean difference	p-value
Respondent's age	0.092	0.024	128	0.141	0.927
Average income	-2.910	0.519	128	-784.509	0.004*
					
	**Physical assault**				
Respondent's age	0.856	0.120	128	1.305	0.393
Average income	-2.167	0.027	128	-592.030	0.032*
					
	**Verbal assault**				
Respondent's age	1.749	0.007	128	2.648	0.083
Average income	-1.342	0.737	128	-371.429	0.182
					
	**Online harassment**				
Respondent's age	1.664	0.087	128	2.521	0.099
Average income	1.456	1.340	128	402.381	0.148

### Association between forms of GBV and other socio-demographic characteristics

Table 3 provides details for all the statistical associations. Considering verbal assault; There was a statistically significant association between the respondents' family structure and their exposure to verbal abuse during the pandemic (p=0.025). Respondents from polygamous homes were about 2 times more likely to be subjected to verbal abuse when compared with those from monogamous homes (Confidence interval: 0.205 to 0.902). There were no statistically significant associations derived between the remaining variables. Respondents that had attained primary/no formal education 2 times more likely to be subjected to verbal abuse compared to those that had at least secondary level of education. In comparison to em-ployed respodents, the unemployed respondents were 2 times more likely to be exposed to verbal abuse. Furthermore, single respondents were about 2 times more likely to be subjected to verbal abuse compared to married/cohabiting respondents.

**Table 3:** Association between respondents' sociodemographic status and GBV

Variables	Adjusted Odds Ratio	p-value	Lower Confidence Interval	Upper Confidence Interval
**Verbal assault**				
Living condition	1.224	0.668	0.487	3.076
Education level	0.468	0.105	0.187	1.171
Occupation	2.044	0.141	0.790	5.288
Marital status	1.964	0.099	0.881	4.378
Family structure	0.430	0.025*	0.205	0.902
**Sexual assault**				
Living condition	0.523	0.165	0.210	1.306
Education level	0.853	0.723	0.355	2.052
Occupation	0.918	0.848	0.384	2.195
Marital status	1.471	0.361	0.671	2.992
Family type	1.235	0.361	0.671	2.497
**Physical assault**				
Living condition	1.202	0.681	0.501	2.885
Education level	1.276	0.580	0.538	3.027
Occupation	0.950	0.909	0.398	2.268
Marital status	1.270	0.526	0.607	2.658
Family type	1.045	0.901	0.521	2.097

Considering sexual and physical assault; there were no statistically significant associations between other socio-demographic variables and the occurrence of sexual and physical assault. However, respondents staying alone were 1.9 times less likely to encounter sexual violence compared to those living with other people. Also, single respondents were about 1.5 times more likely to encounter sexual assault compared to married/cohabiting respondents. Single respondents and respondents that had attained at least secondary education were about 1.3 times more likely to encounter physical assault when compared to married/cohabiting respondents and respondents that had primary/informal education, respectively.

## Discussion

It has been established that poverty is one of the most significant factors that predispose women and girls to GBV 16,17. The low-income status of the study location was further verified via the data about their monthly income; the highest monthly income recorded by the respondents was roughly USD 16. This is no surprise as about half of the Nigerian population has been reported to be in severe poverty, making it the worlds' poverty capital 18,19. The financial hardship is even direr for females in Nigeria because the local social constructs are built to make them dependent on men. Nigerian women have a lower likelihood of being engaged in the labour market and a higher likelihood of being involved in low-paying employments like informal jobs/businesses 20. Majority of the respondents had informal employments such as hairdressing, fashion designing, and baking, while half of the respondents had not attained beyond primary education. Past studies have established causal links between GBV and sociodemographic variables like income level and educational status. For example, a survey that exam-ined the prevalence of domestic violence in Northern Nigeria revealed that women with higher levels of education and those involved with active economic activities or job engagements were less likely to be victims of domestic violence 21. In this study, respondents that had been subjected to sexual (p= 0.004) and physical assault (p=0.032) had a significantly lower average monthly income.

The high prevalence of sexual assault (54.6%), physical assault (52.3%), and verbal assault (41.5%) reported by the respondents within just the first 3 months of the COVID-19 restriction were in tandem with the situation in other countries. Globally, around quarter of a billion females between 15 – 49 years were subjected to sexual and physical assault during the lockdown 22. There were increased frequencies in the number of calls made to domestic violence reporting hotlines in several countries like Singapore, Ukraine, Cyprus, Malawi, Liberia amongst others since the pandemic lockdowns began 23–25. A report from the United Nations Population Fund (UNFPA) revealed that the restrictions put in place to tackle the pandemic significantly increase the exposure of females to GBV, particularly among women and girls in impro-vised and remote settlements 26. A similar situation was observed during the Ebola epidemic in the Democratic Republic of Congo, where there were increased incidences of physical and sexual assault among women and girls during the outbreak 27.

Online GBV has been reported to be an emerging social menace in Nigeria that has resulted in cases of physical and sexual assault, psychological and financial abuse, and even death among young females 1. This makes the 45.4% prevalence of online sexual harassment recorded in this study bothersome. Furthermore, the incidence of FGM reported by about 1 in every 2 females is similar to the regional prevalence of 47.5% reported in Southwestern Nigeria 28. Due to the concerted efforts by non-Governmental Organizations to eradicate FGM in Nigeria, there has reportedly been a decline in the heinous act. However, the persistent absence of Federal law banning FGM makes the achievement of 100% eradication seem farfetched 29.

Furthermore, the fact that less than one-third of the GBV victims reported to the police and that almost half of the respondents were unaware of any local structure available to protect girls and women shows there is still a wide help-seeking gap among victims. Past studies have also revealed that a significant portion of women subjected to GBV do not seek care, which has made the level of help-seeking behaviour in Nigeria comparatively lower than most other countries 28. A Nigeria Demographic Health Survey revealed that around 45% of women subjected to GBV never informed anyone or sought care in any way, while less than a third sought help 28. Another study reported that less than half of women that had been subjected to sexual and physical violence sought care afterwards. It was revealed that most communities lacked an enabling environment and necessary facilities to manage cases of GBV 10. GBV victims generally have little information concerning available structures to tackle such challenges 24. The stigmatization of GBV victims and the inadequacies on the part of health providers and the police have been identified as the major reasons why most girls and women do not seek help or justice after going through such violence 2,28,30. In this study, the most prominent reasons respondents did not report to the police were the lack of trust in the authorities and fear of stigmatization. This proves that the environment is still not enabling enough adequately to protect the rights of our girls and women.

## Conclusion

Results from this survey further backs the hypothesis that women and girls, particularly those in socio-economically disadvantaged situations are vulnerable to GBV during peril periods like a pandemic. The subjection of over 1 in 2 females to at least a form of GBV within 3 months after the lockdown was enacted shows how dire the situation is. It is also clear that there is still a wide gap in the help-seeking behaviour of victims due to their unawareness about/lack of societal support structures, lack of faith in the authorities, and fear of stigmatization. This calls for proactive measures to be taken at the communi-ty, local, state, and national levels of governance in a bid to protect girls and women in underserved communities, especially in times of societal and financial disruption.

## Recommendations

1. Local support structures like women societies, religious leaders, and community security operatives need to be empowered with the knowledge and resources to tackle and address forms of GBV within their locality.

2. There needs to be increased awareness about GBV at the community level. This should be aimed at curtailing the victim-blaming narrative and making information related support structures (such as hotlines) more readily available via local conspicuous posters.

3. Sustainable long-term solutions can be enhanced by setting up structures to erase gender inequalities in education and the labour market.

4. Proactive schemes to protect girls and women from GBV during times of social and economic disruption need to be incorporated in the protocols for disaster management and preparedness.
